# Supplementary material for: An In Vitro Oxidative Stress Model of the Human Inner Ear Using Human-Induced Pluripotent Stem Cell-Derived Otic Progenitor Cells
Source: Antioxidants (Basel). 2024 Nov 16;13(11):1407. doi: 10.3390/antiox13111407 (PMC11591063; doi:10.3390/antiox13111407)
Supplement: Supplementary file 1 [file antioxidants-13-01407-s001.zip › antioxidants-3263540-supplementary.pdf]

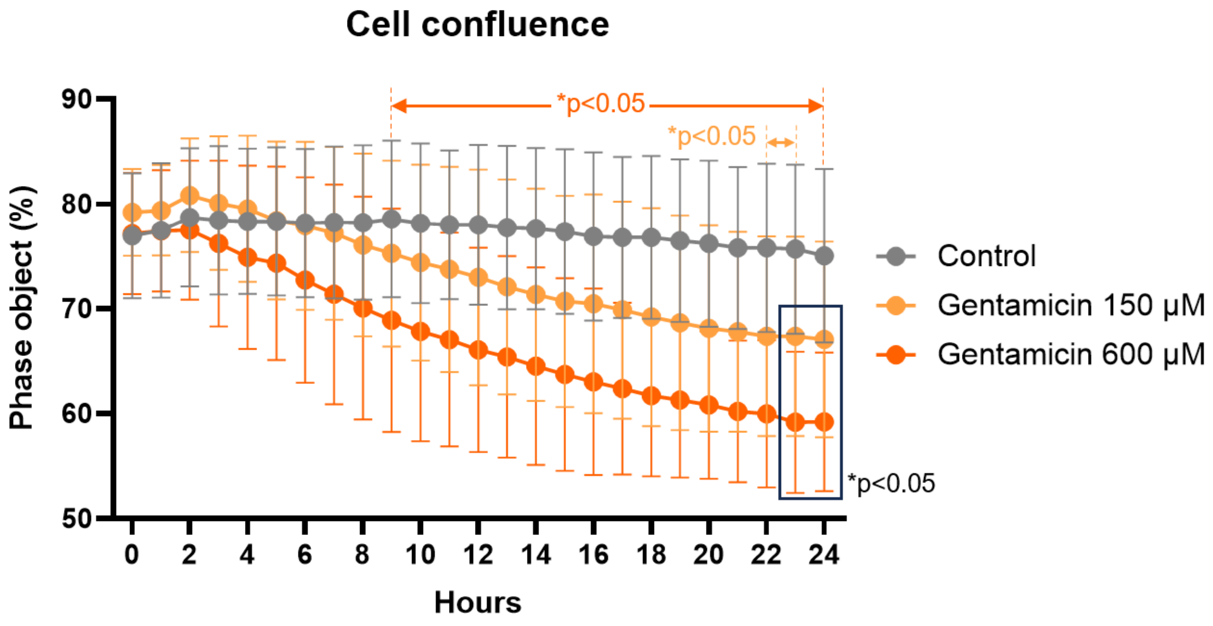

Figure S1. Effects of gentamicin on OPC confluency. Real-time phase contrast measurements of cell confluency under three conditions: 0 µM (vehicle, gray circles), 150 µM (light orange circles), and 600 µM gentamicin (orange circles). Data represent the mean of 3 independent experiments, each with 3 technical replicates per time point ± SD.
